# Three *TaFAR* genes function in the biosynthesis of primary alcohols and the response to abiotic stresses in *Triticum aestivum*

**DOI:** 10.1038/srep25008

**Published:** 2016-04-26

**Authors:** Meiling Wang, Yong Wang, Hongqi Wu, Jing Xu, Tingting Li, Daniela Hegebarth, Reinhard Jetter, Letian Chen, Zhonghua Wang

**Affiliations:** 1 State Key Laboratory of Crop Stress Biology for Arid Areas, College of Agronomy, Northwest A&F University, Yangling, Shaanxi 712100, China; 2 Department of Botany, University of British Columbia, Vancouver, British Columbia, V6T 1Z4, Canada; 3 Department of Chemistry, University of British Columbia, Vancouver, British Columbia, V6T 1Z1, Canada; 4 State Key Laboratory for Conservation and Utilization of Subtropical Agro-bioresources, College of Life Sciences, South China Agricultural University, Guangzhou 510642, China

## Abstract

Cuticular waxes play crucial roles in protecting plants against biotic and abiotic stresses. They are complex mixtures of very-long-chain fatty acids and their derivatives, including C20–C32 fatty alcohols. Here, we report the identification of 32 *FAR*-like genes and the detailed characterization of *TaFAR2*, *TaFAR3 and TaFAR4*, wax biosynthetic genes encoding fatty acyl-coenzyme A reductase (FAR) in wheat leaf cuticle. Heterologous expression of the three TaFARs in wild-type yeast and mutated yeast showed that TaFAR2, TaFAR3 and TaFAR4 were predominantly responsible for the accumulation of C18:0, C28:0 and C24:0 primary alcohols, respectively. Transgenic expression of the three TaFARs in tomato fruit and Arabidopsis *cer4* mutant led to increased production of C22:0–C30:0 primary alcohols. GFP-fusion protein injection assay showed that the three encoded TaFAR proteins were localized to the endoplasmic reticulum (ER), the site of wax biosynthesis. The transcriptional expression of the three *TaFAR* genes was induced by cold, salt, drought and ABA. Low air humidity led to increased expression of *TaFAR* genes and elevated wax accumulation in wheat leaves. Collectively, these data suggest that *TaFAR2*, *TaFAR3* and *TaFAR4* encode active alcohol-forming FARs involved in the synthesis of primary alcohol in wheat leaf and the response to environmental stresses.

Cuticular wax is a major component of cuticle, covering the outermost surfaces of terrestrial plants. It serves mainly as a waterproof barrier, restraining uncontrolled nonstomatal water loss in plants^1^,^2^. Cuticular wax also protects plants against excess UV radiation, bacterial and fungal pathogens as well as insects^3^,^4^,^5^,^6^. Cuticular waxes are complex mixtures of hydrophobic lipids, consisting mostly of very-long-chain fatty acids (VLCFAs, C20 to C34) and their derivatives, including alcohols, aldehydes, alkanes, ketones, and wax esters^7^,^8^,^9^. The wax composition varies greatly among different species and different organs, as well as during plant development. A variety of environmental factors, such as light, temperature and moisture, also influence wax composition considerably^8^,^10^.

The biosynthesis of wax is a complicated process, beginning with the *de novo* C16 or C18 fatty acid synthesis on the outer membrane in the plastid of epidermal cells. The resulting C16 and C18 fatty acyl-CoAs are then elongated to VLCFA wax precursors by a repeating reaction process via fatty acid elongase (FAE) complex in the endoplasmic reticulum (ER)^8^,^11^. Following elongation, wax components are finally produced by converting long-chain fatty acyl-CoAs via two different pathways: the acyl-reduction pathway, producing primary alcohols and wax esters^8^, and the decarbonylation pathway, generating aldehydes, alkanes, secondary alcohols, and ketones^12^. The biosynthesis of primary alcohols, major wax components being found in a wide range of plant species, is completed by acyl-reduction pathway, in which fatty acyl-CoAs are converted into primary alcohols by fatty acyl-CoA reductase (FAR).

In wheat, leaf cuticular waxes consist mainly of primary alcohols, diketones and alkanes, with primary alcohols accounting for up to 86% of the total wax load. C28 primary alcohol has been found to be a major alcohol in wheat leaf of all developmental stages^13^,^14^,^15^,^16^. However, our understanding of molecular mechanism underlying primary alcohol biosynthesis in wheat remains limited. To date, only three genes, *TaFAR1*, *TaFAR5* and *TaTAA1a*, have been identified encoding alcohol-forming FAR in wheat. *TaTAA1a*, an anther-specific gene, encodes FAR related to pollen fertility^17^. TaFAR1 and TaFAR5, two homologs of Arabidopsis CER4, are associated with primary alcohol biosynthesis in wheat anther and leaf cuticle^14^,^15^. However, they appear to mainly produce C22 primary alcohol in yeast, and no more precise proofs reveal that TaFAR1 and TaFAR5 are required for C28 primary alcohol synthesis in wheat. It may present a problem because the bulk of cuticular waxes in wheat leaves are made up of C28 alcohol. Therefore, there may be other unknown FARs, but how many FARs in total are present in wheat leaf? And how many of them are primarily responsible for the generation of C28 primary alcohol in wheat?

The objective of our present work was to identify FARs involved in the biosynthesis of wax primary alcohols in wheat leaf. We identified 32 *FAR*-like genes and characterized in detail the three genes, *TaFAR2*, *TaFAR3* and *TaFAR4*, which encode alcohol-forming FARs in leaf cuticle of wheat. We also examined the responses of these genes to environmental factors. Evidence is presented for the role of TaFAR2, TaFAR3 and TaFAR4 in wax primary alcohol formation in leaf epidermis and the involvement of the three *TaFAR* genes in response to environmental stimuli. In particular, evidence is provided for the major involvement of TaFAR3 and TaFAR4 in the biosynthesis of C28 and C24 primary alcohols, respectively. The present work helps enrich our understanding of the network of wax biosynthesis in plant and provides insights into the modification of cuticle properties to improve crop performance under environmental stresses.

## Results

### Carbon chain length distribution of primary alcohols in wheat

**Figure 1:**
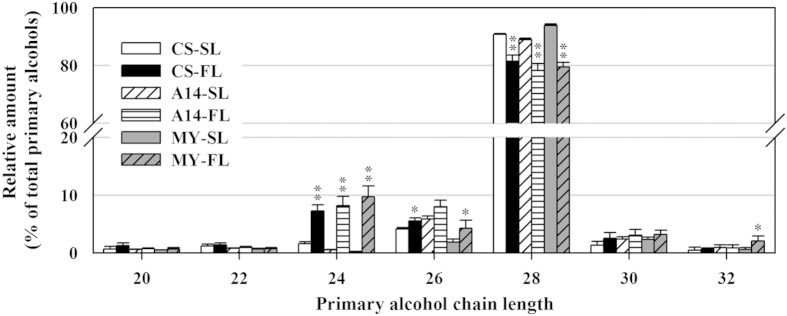
Profile of primary alcohols in seedling and heading leaves of wheat. The content of individual chain length of primary alcohol in seedling leaves (SL) and flag leaves (FL) of three cultivars is shown as relative amount of total primary alcohols (%). Values are means from three replicates. Error bars indicate SD, and significant differences were assessed according to Student’s t-test (* for *P* < 0.05; ** for *P* < 0.01).

In this study, we chose three wheat cultivars which showed obvious difference in accumulation and micromorphology of leaf cuticular wax (Supplementary Fig. S1 and Table S1). Primary alcohols were the dominant wax components, accounting for 82–86% and 40–62% of wax coverage in seedling and flag leaves, respectively. Among primary alcohols found in wheat leaf blade, C28 primary alcohol was the most abundant one (Supplementary Table S1). Our results showed that chain length distributions within the class of primary alcohols changed along with developmental stages in wheat (Fig. 1). We found a significant decrease of C28 primary alcohol and an obvious increase of C24 primary alcohol in flag leaves compared with that in seedling leaves. These results suggest that change in the chain length distributions of primary alcohols might be due to the altered expression of alcohol-forming FARs at different developmental stages.

### Identification of *FAR* genes in wheat

**Figure 2:**
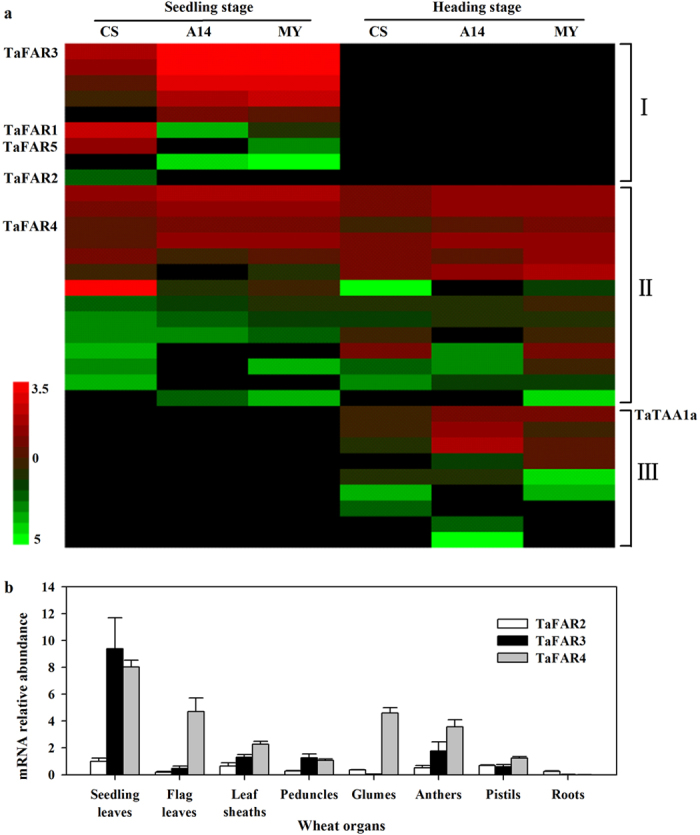
The expression of *FAR* genes in wheat leaves. (**a**) Heat map showing the expression of 32 *FAR* genes at different developmental stages. The relative expression levels (Log2) of alcohol-forming *FAR*s from seedling and flag leaves of three wheat cultivars are shown by transcriptome sequencing. Red and green indicate high and low expression levels, respectively. The accession numbers of 32 *FARs* are summarized in Supplementary Table S2. (**b**) Tissue-specific expression analysis of *TaFAR2*, *TaFAR3* and *TaFAR4* by qRT-PCR. Each value is the mean of three independent parallel experiments. Error bars = SD.

Our results have shown that primary alcohols were the key components of cuticular waxes and the profiles of primary alcohols varied at different growth stages. The next goal in this study was to obtain genes responsible for the production of primary alcohols in wheat leaf cuticle. To achieve this goal, a BLAST search of the wheat database (Ensembl Plants) was performed using the amino acid sequence of Arabidopsis CER4 (GenBank accession no. NP_567936). In total, 32 *FAR*-like genes expressing in leaves were screened out after comparing retrieved sequences with our transcriptome data (Supplementary Table S2). Based on expression patterns in leaves, these genes can be categorized into three groups: I) seedling leaf-specific expression, II) seedling and flag leaf-specific expression and III), flag leaf-specific expression (Fig. 2a). Of 32 *FAR* genes, 9 genes were expressed in seedling leaves, 9 genes were expressed in flag leaves, and 14 genes were expressed in both seedling and flag leaves. This result confirmed that there were multiple *FAR* genes in wheat leaves, and different *FAR* genes were expressed differentially according to growth stages.

Further analysis showed that highly expressed genes were mainly concentrated in the first and second groups, while genes with relatively low expression were in group III. Thus, we focused our subsequent analyses on the genes in groups I and II. Among 9 genes expressed in seedling leaves, *TaFAR1* and *TaFAR5* genes have been shown to be mainly responsible for the production of C22 primary alcohol in yeast^14^,^15^. One gene (No. 1), designated as *TaFAR3*, was highly expressed in seedling leaves of all three tested cultivars, while another gene (No. 9), designated as *TaFAR2*, was only expressed in Chinese Spring. A gene (No. 12), designated as *TaFAR4*, was highly expressed in both seedling and flag leaves of all three cultivars (Supplementary Table S2 and Fig. 2a). Of the 32 putative FAR-like genes, 11 genes were on A genome, 9 on B genome and 12 on D genome. Further examination showed that *TaFAR2*, *TaFAR3* and *TaFAR4* genes were on chromosome 7DS, 4BS and 5AL, respectively (Supplementary Table S2). Two genes, Traes_7AS_3FFD661DA.1 (No. 30) and Traes_4AL_2475C298D.2 (No. 5), orthologous to *TaFAR2* and *TaFAR3,* were on 7AS and on chromosome 4AL, respectively.

Tissue-specific expression patterns of the three *TaFAR* genes were determined by quantitative real-time PCR. We detected *TaFAR2*, *TaFAR3* and *TaFAR4* transcript levels in various organs, including seedling leaves, flag leaves, leaf sheaths, peduncles, glumes, anthers, pistils and young roots. Our results showed that *TaFARs* were widely expressed, with the most notable accumulation being in aerial organs and very low levels of accumulation in underground organ (root, Fig. 2b). Further detailed analysis showed that *TaFAR3* is mainly expressed in seedling leaves, while *TaFAR4* was abundantly expressed in seedling and flag leaves, with considerable transcript accumulation being also detected in glumes and anthers. These results are consistent with our transcriptome results, indicating the significant role of *TaFARs* in the accumulation of leaf cuticular wax at seedling stage.

### Bioinformatics analysis of TaFAR2, TaFAR3 and TaFAR4

Sequence analysis revealed that *TaFAR2*, *TaFAR3* and *TaFAR4* cDNA contains an open reading frame of 1,485, 1,494 and 1,524 bp, encoding a polypeptide of 494, 497 and 507 amino acids, respectively. Prokaryotic expression revealed that His-TaFAR2, His-TaFAR3 and His-TaFAR4 fusion proteins were 60.2, 60.2 and 60.8 kDa, in agreement with the predicted size of 56.3, 55.9 and 57.2 kDa, respectively (Supplementary Fig. S2).

**Figure 3:**
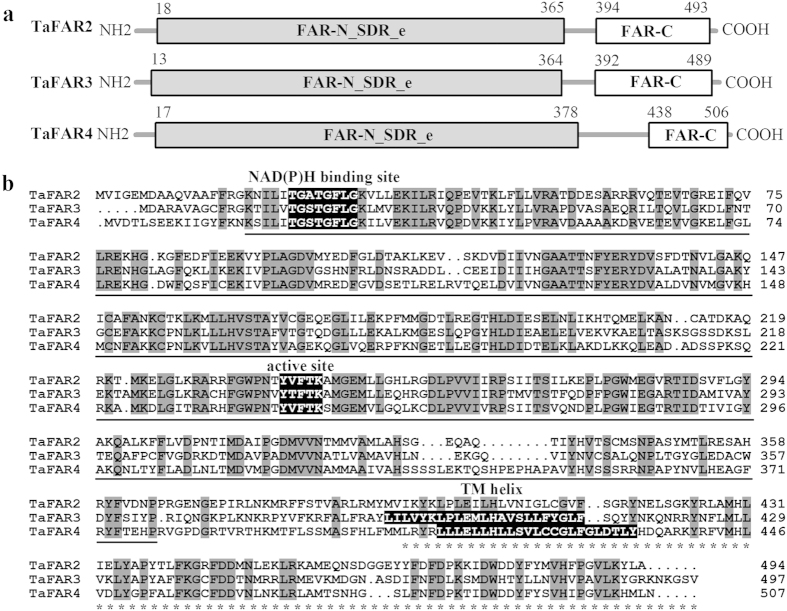
A schematic diagram illustrating the structure and sequence of the three TaFAR proteins. (**a**) Predicted functional domains. The conserved FAR-N_SDR_e and FAR_C domains are marked by underline and asterisks, respectively. The numbers indicate amino acid positions. (**b**) Amino acid sequences of the three TaFARs. Identical amino acids are highlighted in dark gray. Three conserved motifs, NAD(P)H binding site motif (TGXXGXXG), active site motif (YXXXK) and TM (transmembrane) helix motif are highlighted in black (where X represents any amino acid). The GenBank accession numbers of FAR sequences are as follows: TaFAR2, KJ675403; TaFAR3, KT963076; TaFAR4, KT963077.

Protein domain searches against the NCBI’s conserved domain database revealed that the three deduced TaFAR proteins contain a Rossmann-fold NADB binding domain (FAR-N_SDR_e) linked with a fatty acyl-CoA reductase domain (FAR_C) at the C-terminal end (Fig. 3a), consistent with FARs from other plant species, such as TaFAR1 from wheat^15^, and FAR5 and FAR8 from Arabidopsis^18^. In addition, the NADB domain has two classical conserved motifs, a NAD(P)H binding site motif (TGXXGXXG) and an active site motif (YXXXK) (Fig. 3b). Analysis by TMHMM program revealed that TaFAR3 and TaFAR4 contain one transmembrane domain between residues 390 to 412 and 411 to 433, respectively. This feature is in agreement with the jojoba FAR, which also contains membrane-spanning domains^19^. Although no transmembrane domain was found in TaFAR2, similar result was reported on an alcohol-forming FAR in Arabidopsis^20^ (Fig. 3b). Based on these analyses, we hypothesized that these three TaFAR proteins possess FAR activity associated with primary alcohol biosynthesis in wheat, and in the following experiments, further evidence was provided to support our hypothesis.

### Heterologous expression of TaFAR2, TaFAR3 and TaFAR4 in yeast

To investigate whether TaFAR2, TaFAR3 and TaFAR4 are fatty-acyl-CoA reductases catalyzing the production of primary alcohols, we expressed the three *TaFAR* genes in yeast. Our work has shown that the main components of wheat leaf cuticular waxes are primary alcohols with carbon chain lengths ranging from C20 to C32. However, the wild-type yeast produces VLCFAs only up to C26 (with traces of C28) and due to this chain length limitation, it could not be possible for TaFARs to catalyze the production of primary alcohols beyond C28^20^,^21^,^22^. To avoid this problem, we used a mutated yeast INVSur4#, which is capable of generating C16 to C32 fatty acids^23^,^24^, to express the three TaFARs (Supplementary Table S3).

**Figure 4:**
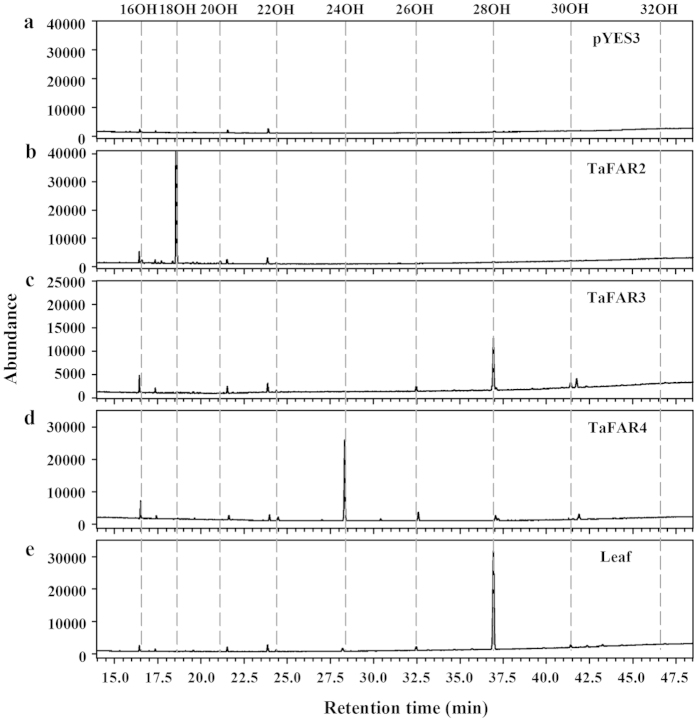
Heterologous expression of the three TaFARs in yeast mutant INVSur4#. The yeast expressing empty vector control pYES3 (**a**) or vector harboring *TaFAR2* (**b**), *TaFAR3* (**c**) or *TaFAR4* (**d**). Transgenic yeast cells were grown in stringent medium lacking trptophan and uracil. Primary alcohols were separated from other neutral lipids by TLC. In empty vector control, no primary alcohols were detected. In contrast, yeast harboring TaFAR2, TaFAR3 and TaFAR4 preferentially produced C18, C28 and C24 primary alcohols, respectively. All primary alcohols were also observed in wheat (cv. Chinese Spring, (**e**) leaf.

GC-MS analysis showed that yeast cells produced novel compounds when TaFARs were expressed in INVSur4#. They were identified as primary alcohols ranging from C16 to C20 in transgenic yeast expressing TaFAR2 (Fig. 4b), C22:0-OH to C30:0-OH when expressing TaFAR3 (Fig. 4c), and C22:0-OH to C26:0-OH when expressing TaFAR4 (Fig. 4d), whereas yeast cells hosting empty vectors accumulated no primary alcohols (Fig. 4a). It should be noted that all these primary alcohols could be detected in wheat leaf where C28:0-OH was the dominant alcohol (Fig. 4e).

Even though the three proteins catalyzed the synthesis of primary alcohols, the preference of each TaFAR for acyl chain length was significantly different. Expression of TaFAR2 resulted in large quantities of C18:0-OH (99.3%), and very small amounts of C16:0-OH (0.5%) and C20:0-OH (0.3%). The major primary alcohol produced when expressing TaFAR4 was C24:0-OH (83.2%), but C22:0-OH (3.7%) and C26:0-OH (13.1%) primary alcohols were also detected. In the case of TaFAR3, the highest production of primary alcohol was C28:0-OH, followed by C26 (5.7%), C22 (2.4%), C30 (1.2%), and C24 (1.0%) alcohols (Supplementary Table S4). It is worth noting that we got the consistent results of chain length distributions of primary alcohols when expressing TaFAR2 and TaFAR4 either in the wild-type yeast (INVSc1) or in the mutated yeast INVSur4#, however, shorter carbon chain lengths (ranging from C22 to C26) were observed when TaFAR3 was expressed in the wild-type yeast, suggesting that TaFAR2 or TaFAR4 is not sufficient for the production of alcohols longer than C26 in yeast (Supplementary Fig. S3). Altogether, these results confirmed that TaFAR2, TaFAR3 and TaFAR4 from wheat indeed have the reductive activity of fatty acyl-coenzyme A reductase, catalyzing the production of long and very-long-chain primary alcohols. Remarkably, TaFAR3 had a major function of producing C28 primary alcohol, which is the richest alcohol species of wheat leaf wax.

### Expression of TaFAR2, TaFAR3 and TaFAR4 results in increased production of primary alcohols in fruit cuticle of tomato

**Figure 5:**
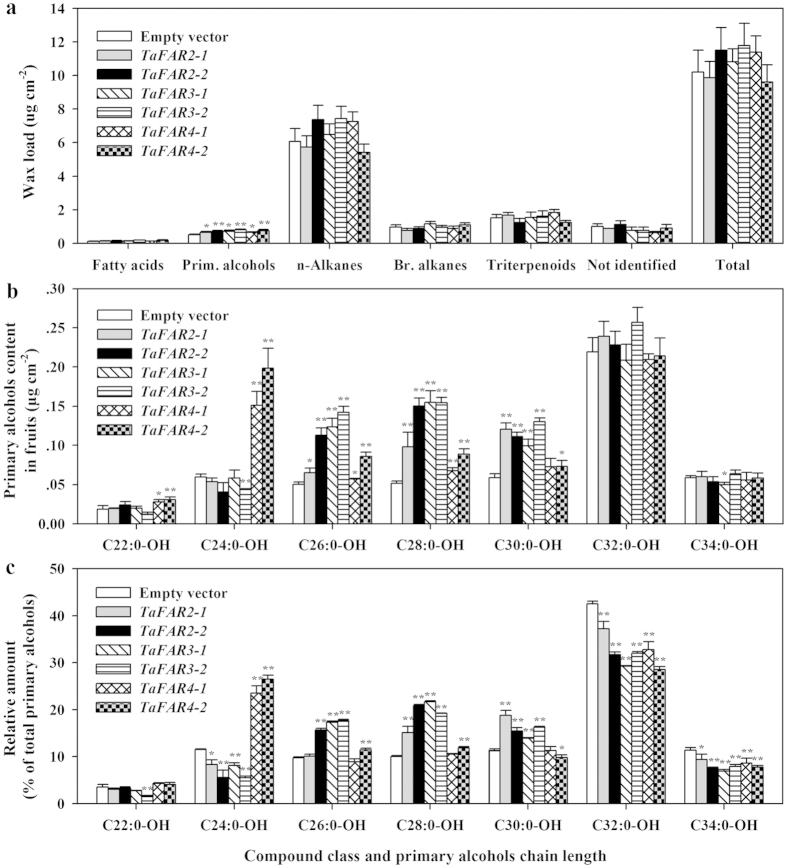
Wax analysis of fruit cuticles from tomato lines expressing TaFAR2, TaFAR3 and TaFAR4. Cuticular wax mixtures were extracted from ripe fruits of T1 generation transgenic lines expressing empty vector or vector harboring *TaFAR2*, *TaFAR3* or *TaFAR4.* (**a**) Total wax amounts and coverage of individual compound classes. Data are expressed as μg per unit cm^2^. (**b**) Absolute amount (μg cm^−2^) of individual primary alcohol class. (**c**) Proportional content (%) of individual primary alcohol class of total primary alcohols load. Prim. alcohols, primary alcohols; Br. alkanes, Branched alkanes. Values are means of n ≥ 3 biological replicates. Error bars = SD. Asterisks represent significant differences between TaFAR-expressors and empty vector control (t-test: * for *P* < 0.05; ** for *P* < 0.01).

To verify that TaFAR2, TaFAR3 and TaFAR4 are alcohol-forming FARs involved in wax biosynthesis in higher plants, we expressed these three genes downstream the 35S promoter in tomato via *Agrobacterium tumefaciens*-mediated transformation. GC-FID analysis of fruit cuticular wax composition and total load of T1 transgenic lines revealed a significant increase in primary alcohols but no obvious change in total wax load. The content of total primary alcohols increased from 0.52 μg cm^−2^ in fruit cuticle of control line to 0.72 μg cm^−2^ in transgenic line TaFAR2-2, 0.80 μg cm^−2^ in line TaFAR3-2 and 0.75 μg cm^−2^ in line TaFAR4-2 (Fig. 5a).

Further analysis of chain length patterns within compound classes revealed that most of the individual wax components in fruit cuticles of transgenic lines stayed almost the same as those in control line. The most obvious variation was found for primary alcohols, with C26, C28 and C30 primary alcohols being increased in transgenic lines TaFAR2 and TaFAR3 (Fig. 5b,c). Similar chain length patterns for primary alcohols in transgenic line TaFAR4 were also found except that the levels of C22 and C24 primary alcohols were also significantly increased compared with control line. Transgenic line TaFAR2-2 showed the greatest increase in the absolute amounts of C26:0-OH, C28:0-OH and C30:0-OH, which was enhanced by 2.2, 2.9 and 1.9 fold, respectively, compared with control line. In transgenic line TaFAR3-2, the absolute amounts of C26:0-OH, C28:0-OH and C30:0-OH was increased by 2.8, 3.0 and 2.2 fold relative to control line. The C22, C24, C26, C28 and C30 primary alcohols were found to be increased by 1.7, 3.3, 1.7, 1.7 and 1.3 fold, respectively, in transgenic line TaFAR4-2 (Fig. 5b). These results from primary alcohol changes provided further evidence that the three TaFARs are alcohol-forming FARs, and that they are responsible for the wax very-long-chain alcohols formation in the epidermal cells of tomato fruits.

### Expression of TaFAR2, TaFAR3 and TaFAR4 in Arabidopsis *cer4-3* mutant

Another experiment was performed for further examination on the role of the three TaFARs in primary alcohol biosynthesis. We expressed *TaFAR2*, *TaFAR3* and *TaFAR4* genes in the Arabidopsis *cer4* mutant defective in the accumulation of C24:0 and C26:0 primary alcohols^20^. The appearance of wax phenotype of TaFAR lines was examined in comparison with the corresponding transgenic lines expressing empty vector in *cer4-3* mutant background. No change was detected by sight or with SEM in transgenic lines, and a relatively smooth stem surface appeared as was in *cer4-3* mutant. We then analyzed the wax profile of stems and leaves in TaFAR lines. Similar to the results obtained from transgenic tomato, no significant difference could be detected in the level of each wax component or the total wax coverage of transgenic lines compared with the empty vector control line, except for an increase of total primary alcohols and slightly lower aldehydes in leaves of TaFAR4 transgenic plants (Supplementary Fig. S4).

**Figure 6:**
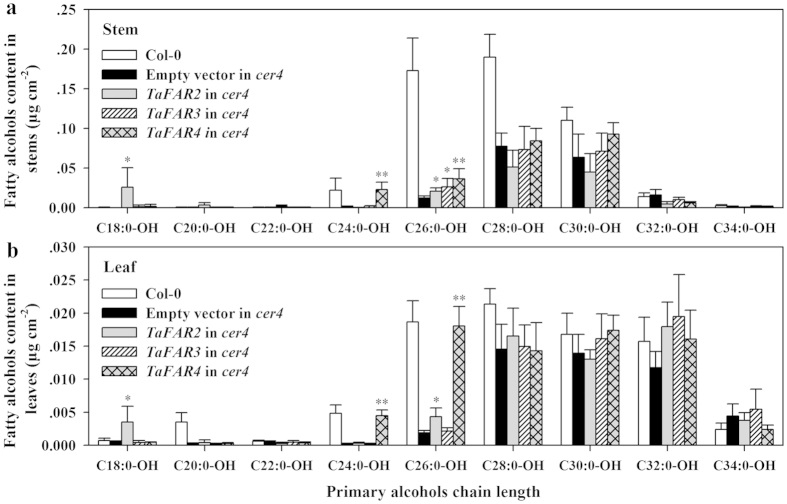
Levels of primary alcohols in *Arabidopsis thaliana* lines expressing TaFAR2, TaFAR3 and TaFAR4. Cuticular wax mixtures were extracted from stems and leaves of wild-type Arabidopsis, and transgenic lines expressing empty vector or vector harboring *TaFAR2*, *TaFAR3* or *TaFAR4* gene in *cer4* mutant background. Primary alcohol contents in stems (**a**) and leaves (**b**) are shown as absolute amount (μg cm^−2^). Each value represents the mean of n ≥ 5 replicates. Error bar = SD. Asterisks indicate significant differences between TaFAR-expressors and empty vector control (t-test: * for *P* < 0.05; ** for *P* < 0.01).

Expectedly, in spite that the total primary alcohol levels were not affected in TaFAR2 and TaFAR3 lines, the amounts of C18:0-OH and C26:0-OH was slightly increased in both leaves and stems in TaFAR2 line, while the amount of C26:0-OH was increased only in stems in TaFAR3 line. In TaFAR4 line, the amounts of C22:0-OH and C24:0-OH, especially the C24:0-OH, were significantly increased in both leaves and stems (Fig. 6a,b). The increase of primary alcohols in Arabidopsis *cer4* mutant confirmed again that the three TaFARs under investigation were involved in synthesis of wax alcohol in plant cuticle.

### Subcellular localization of the three TaFARs

**Figure 7:**
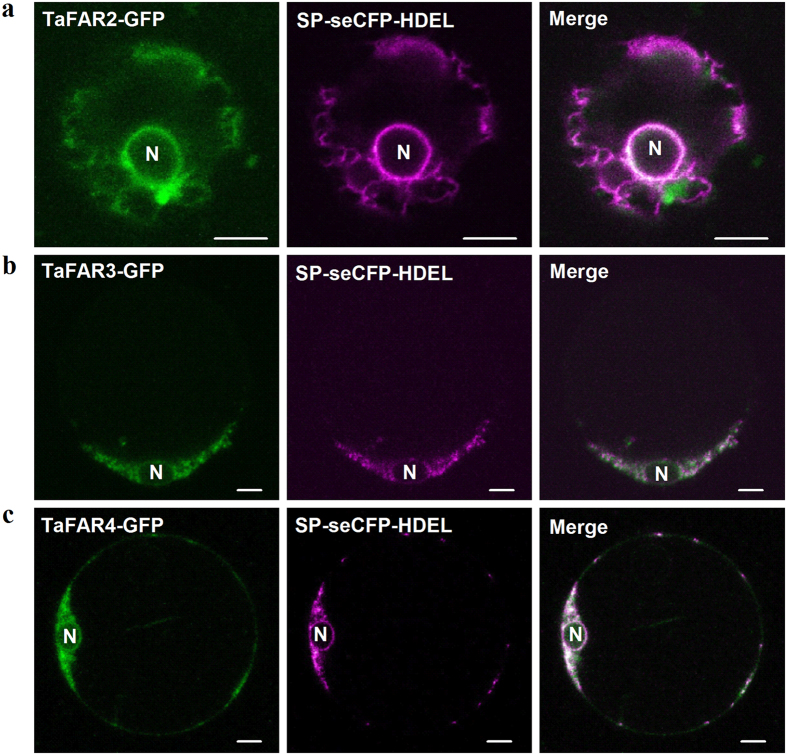
Subcellular localization of TaFAR2, TaFAR3 and TaFAR4 proteins in rice protoplast. TaFAR2-GFP (**a**), TaFAR3-GFP (**b**) or TaFAR4-GFP (**c**) fusion protein was colocalized predominantly (left panel) with the ER marker SP-seCFP-HDEL (middle panel) in the merge (right panel). SP-seCFP-HDEL is a CFP fused ER marker. N, nucleus. Bars = 5 μm.

Previous studies have proved that major enzymes involved in the biosynthesis of primary alcohols and other wax components are located to the ER in plant epidermis cell^7^,^8^, thus, we predicted that the three TaFAR proteins with the function of primary alcohol synthesis also localize to the ER. To determine if this is indeed the case, the intracellular localization of TaFAR2, TaFAR3 and TaFAR4 proteins were carried out in rice leaf protoplasts expressing *TaFARs* cDNA C-terminally tagged with green fluorescent protein (GFP) under the control of the CaMV 35S promoter. A known ER marker, SP-seCFP-HDEL, tagged with cyan fluorescent protein (CFP) was co-transformed into rice leaf protoplasts. The GFP signals for TaFAR2 (Fig. 7a), TaFAR3 (Fig. 7b) and TaFAR4 (Fig. 7c) fusion proteins were detected in the same subcellular compartments with CFP signals, showing a typical ER pattern. As expected, the confocal microscopy clearly displayed that TaFAR2-GFP, TaFAR3-GFP and TaFAR4-GFP fusion protein co-localized with the ER marker, surely indicating the three TaFARs reside in the ER where wax biosynthesis occurs.

### Induction of *TaFAR2*, *TaFAR3* and *TaFAR4* genes

**Figure 8:**
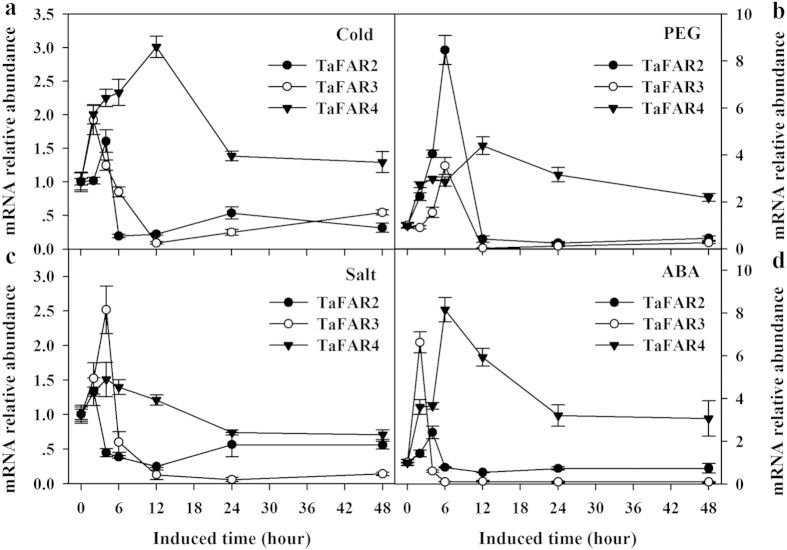
Induction of *TaFAR1*, *TaFAR3* and *TaFAR4* expression by various stresses. Four-week old Chinese Spring plants were exposed to subsequent treatments. (**a**) Cold stress: 4 °C. (**b**) PEG stress: 20% (w/v) PEG6000. (**c**) Salt stress: 200 mM NaCl. (**d**) ABA treatment: 100 μM ABA. The mRNA relative abundance was detected by qRT-PCR. Each value is the mean of three independent parallel experiments. Error bars represent the SD.

Cuticular wax accumulation can be influenced by a series of environmental stresses^4^,^15^,^22^,^25^. Therefore, we asked whether the selected wax biosynthetic genes are responsive to environmental factors. In this study, we examined the expression of endogenous *TaFAR2*, *TaFAR3* and *TaFAR4* in wheat seedlings in response to cold, PEG-induced drought, salt and ABA hormone treatment. The transcripts of all three *TaFARs* were affected by stress treatments. The transcripts of *TaFAR4* peaked at 12 h in response to cold treatment (Fig. 8a), while those of *TaFAR2* and *TaFAR3* peaked at 4 h and 2 h, respectively. After being treated by PEG6000, *TaFAR2* and *TaFAR3* showed the highest expression level at 6 h, whereas *TaFAR4* at 12 h (Fig. 8b). Under NaCl-induced salt stress, all three genes showed a rapid induction, reaching peaks at 2 h (*TaFAR2* and *TaFAR3*) and 4 h (Fig. 8c). We also exposed wheat seedlings to ABA, a signaling molecule thought to induce wax biosynthesis. The highest transcript abundance occurred at 4 h for *TaFAR2* and 2 h for *TaFAR3*, and at 6 h and remained high after 48 h for *TaFAR4* (Fig. 8d). These results showed that the three *TaFAR* genes could be up-regulated by ABA and abiotic stresses.

### Influence of air humidity on *TaFARs* expression and cuticular wax accumulation

**Figure 9:**
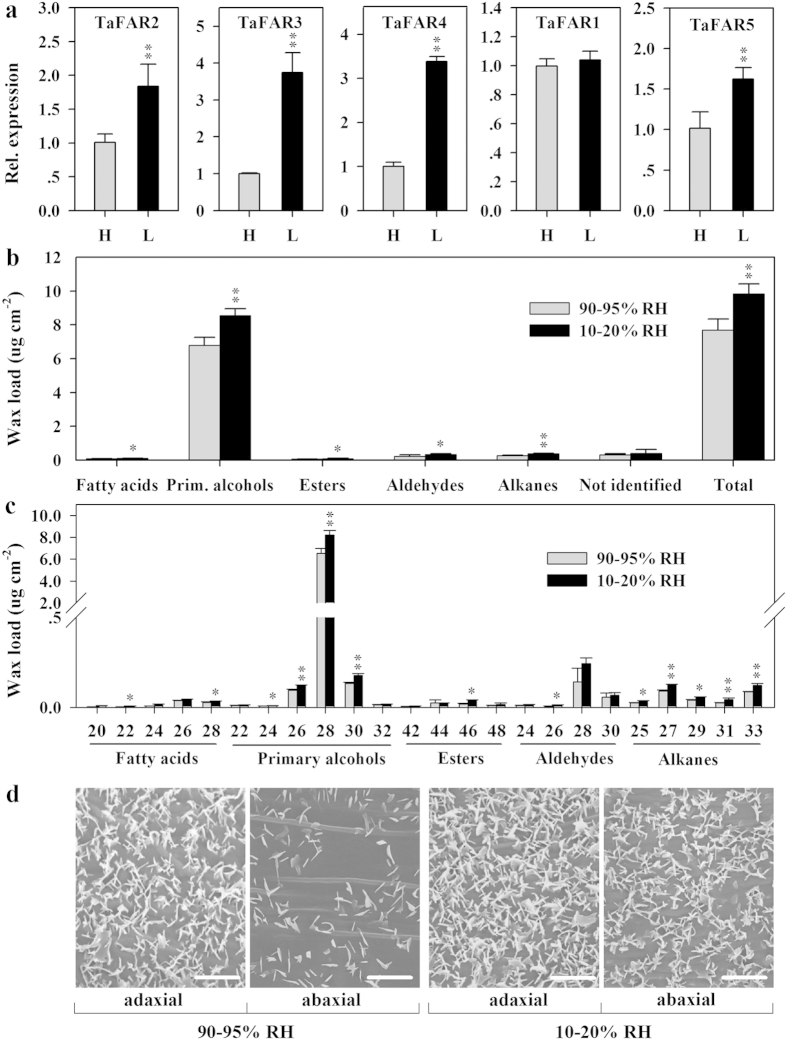
Effects of air humidity on epicuticular wax deposition. Three-week old Chinese Spring seedlings grown under low (10–20% RH) and high (90–95% RH) humidity were used for subsequent analysis. (**a**) Relative expression analysis of *TaFARs* by qRT-PCR. H, high (90–95% RH) humidity; L, low (10–20% RH) humidity. (**b,c**) The cuticular wax composition and chain length distribution within compound classes. All values represent means of three independent parallel experiments. Prim. alcohols, primary alcohols. Error bars indicate SD and asterisks represent significant differences (t-test: * for *P* < 0.05; ** for *P* < 0.01). (**d**) Elevation of epicuticular wax crystals after low humidity treatment. Wax crystals on adaxial and abaxial surfaces of wheat leaves were detected by SEM at 10,000X magnification. Bars = 2 μm.

To examine more directly the role of the three *TaFARs* in cuticular wax biosynthesis and in drought stress response, we investigated the effects of air humidity on *TaFARs* expression and cuticular wax accumulation. To this end, Chinese Spring seedlings were exposed to either low air humidity (10–20% RH) or high air humidity (90–95% RH). Under low humid condition, expression of *TaFAR2*, *TaFAR3*, *TaFAR4* and *TaFAR5* was significantly up-regulated (Fig. 9a). Thus, we expected an increase in leaf wax accumulation in plants growing under low air humidity. We found that the amounts of leaf waxes were elevated by 28%, including a 26% significant increase in primary alcohols, compared with those under high humidity (Fig. 9b,c). In addition, SEM analyses showed that the deposition of epicuticular wax crystals was affected by different humid growth conditions. A dense array of platelet-shaped wax crystals was found covering leaf surfaces exposed to low humidity, while leaves grown under high humidity were covered with scattered crystals (Fig. 9d). These observations indicate that the cuticular wax accumulation is partly contributed by low-humidity induction of *TaFAR* genes.

## Discussion

In this study, we identified and characterized *TaFAR2*, *TaFAR3* and *TaFAR4* genes that encode fatty acyl-coenzyme A reductase (FAR) in wheat. We concluded that *TaFAR2*, *TaFAR3* and *TaFAR4* are involved in the production of primary fatty alcohols in wheat leaves, that TaFAR3 and TaFAR4 largely contribute to the biosynthesis of C28:0-OH and C24:0-OH, respectively, and that the three *TaFAR* genes are responsive to environmental stress.

We first performed an experiment to express TaFAR2, TaFAR3 and TaFAR4 in yeast in order to validate that the three TaFARs are involved in primary alcohol biosynthesis. Expression in yeast confirmed that TaFAR2, TaFAR3 and TaFAR4 produce primary alcohols ranging from C16:0 to C30:0. It is worth noting that TaFAR3 is primarily responsible for producing C28 primary alcohol, which is the dominant wax component in wheat leaf. The production of C28:0-OH has never been observed in heterologous expression of FARs from other plant species in yeast^22^,^26^. Arabidopsis CER4/FAR3 and wheat TaFAR1 and TaFAR5 are able to generate C28 primary alcohol *in planta*, however, they fail to produce C28 primary alcohol in yeast system^14^,^15^,^20^. The failure might be due to either the unavailability of appropriate substrates or the lack of substrate specificities of these FARs in yeast. In our experiment, we mutated yeast in order to produce substrates with a wider range of chain lengths. Expression of TaFAR2 and TaFAR4 in wild-type yeast resulted in the production of primary alcohols of less than 28 carbons, yet these two TaFARs were still unable to make primary alcohols of C28 or longer even when substrates with diverse chain lengths were available in our mutated yeast system, indicating that TaFAR2 and TaFAR4 are incapable of catalyzing substrates with chain length past C26 in yeast (Fig. 4 and Supplementary Fig. S3). In contrast, the expression of TaFAR3 in wild-type yeast led to the accumulation of primary alcohols with maximum chain length of 26 carbons, but when expressed in mutated yeast, TaFAR3 predominantly generated C28 primary alcohol. These results imply that TaFAR3 has wider substrate range than TaFAR2 and TaFAR4, and prefers C28 substrate to other chain lengths of substrates in yeast system.

We then carried out another two experiments *in planta* to gain further insights into the role of TaFAR2, TaFAR3 and TaFAR4 in primary alcohol biosynthesis in higher plants. Transgenic expression of the three TaFARs led to increased accumulation of C22–C30 primary alcohols in fruit cuticles of tomato, suggesting that TaFAR2, TaFAR3 and TaFAR4 are involved in the synthesis of wax primary alcohols. The molecular complementation of Arabidopsis *cer4* mutant with TaFAR2, TaFAR3 and TaFAR4 partially restored the function of CER4, which is mainly responsible for the production of C24 and C26 primary alcohols in Arabidopsis, further supporting that TaFAR2, TaFAR3 and TaFAR4 are alcohol-forming FARs. However, *in planta* expression of each of the three TaFARs led to significant accumulation of more than one primary alcohol, which is different from yeast expression producing only one major alcohol. It seems that each of the three TaFARs has strong preference for a wide range of chain lengths of substrates in planta rather than just one chain length of substrate in yeast. We are aware that in many cases, the substrate specificities of FARs are not strictly consistent between yeast and plant^14^,^15^,^20^. This inconsistency raises the question: What governs chain length specificities of TaFARs and what causes the difference in specificities between yeast and plant? It has been shown that FAR chain length specificity is determined by specific amino acids at the junction of the Rossmann-fold domain and the FAR_C domain^18^. If this is also the case for TaFARs, then it is possible that the key amino acids in TaFAR proteins undergo certain types of modifications which change chain length specificities and stabilities in yeast or plant. Further work needs to be done to find out what amino acid residues are important for conferring TaFAR substrate specificities and how specificities are impacted in plant cell.

FAR enzymes that produce fatty alcohols, and FAE complex that generate wax precursors, as well as other reported wax biosynthetic enzymes, such as TaFAR5 in wheat^14^, GL8 in maize^27^, CER1 in Arabidopsis^24^ and CsWAX2 in cucumber^28^ all reside in ER membranes. Likewise, we anticipated that the three TaFARs would localize to the ER if they were involved in the generation of primary alcohols. Intracellular localization of the three TaFARs in rice leaf protoplasts revealed that these wheat TaFARs also reside in the ER. Moreover, membrane-spanning domain was found in TaFAR3 and TaFAR4 proteins. However, no transmembrane domain was found in TaFAR2 protein, suggesting TaFAR2 may associate with ER membrane through other means. FAR functions subsequent of fatty acid elongation and enzymes catalyzing elongation have been demonstrated to be localized to the ER. Hence, the ER localization of TaFAR2, TaFAR3 and TaFAR4 is compatible with their biochemical functions as enzymes reducing fatty acyl-CoAs to primary alcohols in wax synthesis.

The chain length distribution of primary alcohols varies greatly among plant species, and in many cases between different organs and/or developmental stages^13^,^15^,^29^,^30^,^31^. The difference in chain length distribution of primary alcohols is due to both the availability of different lengths of substrates (fatty acids) and the expression of diverse FARs that confer different substrate specificities. In our research, the successful generation of C28 primary alcohol in yeast mutant expressing TaFAR3 is ascribed to, in addition to TaFAR3 specificity, the production of C28 fatty acid. FAR preference for different chain lengths of substrates can also explain the difference in chain length distribution of primary alcohols. TaFAR3 largely yielding C28 primary alcohol is highly expressed in seedling leaves, while TaFAR1, TaFAR2 and TaFAR5 mainly producing primary alcohols shorter than 28 carbons are expressed at very low levels at seedling stage. The expression difference of TaFAR3 partly explains why C28 primary alcohol is the dominant primary alcohol in leaf of wheat seedlings. TaFAR4 with high expression in leaf at seedling stage also expressed highly in flag leaves at heading stage, whereas TaFAR1, TaFAR2, TaFAR3 and TaFAR5 barely expressed in flag leaves. Given that TaFAR4 is mainly involved in producing C24 primary alcohol, it is possible that TaFAR4 contributes to the increased percentage content of C24 primary alcohols in flag leaves. The expression of the three TaFARs with distinct catalytic preference leads to the developmental difference in primary alcohol chain length distribution of wheat.

Waxes are proved to play multiple roles in protecting plants against adverse growth conditions. One key function of waxes is to prevent nonstomatal water loss, which is crucial for plant survival under drought conditions^25^,^32^. Previous studies have shown that wax biosynthesis-related genes, such as *FAR4*, *KCS6* and CER1 in Arabidopsis, can be induced by drought stress, and thus the accumulation and composition of cuticular wax are influenced^22^,^33^,^34^. As a drought signal, ABA has been demonstrated to be necessary to activate a set of cuticle-associated genes^25^,^35^. In wheat leaves, primary alcohols are major components of wax, so we expect that primary alcohol biosynthetic genes are responsive to environmental stimuli. In this study, all four tested stimuli induced the expression of *TaFARs* at the transcriptional level (Figs 8 and 9). Furthermore, low air humidity induced the expression of *TaFARs* and resulted in increased accumulation of leaf cuticular wax, indicating that the up-regulated expression of *TaFARs* is an important adaptation strategy that minimizes cellular and organismal dehydration in plants under drought conditions.

In summary, we have obtained *TaFAR2*, *TaFAR3* and *TaFAR4* genes from wheat leaf and provided evidence that the three genes encode ER-localized FARs involved in the production of primary alcohols in cuticular wax biosynthesis. TaFAR3 is dominantly responsible for catalyzing the production of C28 alcohol, which is a major cuticular wax component found in wheat leaves, while TaFAR4 is mainly involved in the generation of C24 alcohol. Furthermore, at the transcriptional level, the expression of *TaFAR2*, *TaFAR3* and *TaFAR4* is highly regulated by abiotic stresses. By controlling primary alcohol formation, *TaFAR2*, *TaFAR3* and *TaFAR4* appear to be key genes involved in cuticular wax metabolism and stress responses in wheat.

## Materials and Methods

### Plant materials and stress treatments

Three hexaploid wheat (*Triticum aestivum* L.) cultivars Chinese Spring (CS), A14 and Mingyou633 (MY) were grown at the experimental farm of Northwest A&F University, Yangling, China, during the 2012 and 2013 wheat-growing seasons. The seedling leaves (SL) and flag leaves (FL) of the three cultivars were used for transcriptome and waxes analysis. Simultaneously, to examine the tissue-specific expression patterns of wheat *TaFARs*, different organs including seedling leaves, flag leaves, leaf sheaths, peduncles, glumes, anthers, pistils and roots from Chinese Spring were harvested for analysis of transcript level.

For analysis of *TaFARs* response to abiotic stresses, four-week-old Chinese Spring seedlings grown on MS-agar media were carefully pulled out and transferred to MS liquid media containing either 100 μM ABA, 200 mM NaCl or 20% (w/v) PEG6000. Cold treatment was conducted by exposure of plants to 4 °C. The duration of all treatments was set at 0 h, 2 h, 4 h, 6 h, 12 h, 24 h and 48 h. Sixty–seventy seedlings were subjected to each treatment and three parallel treatments were carried out for each treatment. The leaves of each time point were harvested for expression analysis of *TaFAR* genes.

For analysis of *TaFARs* expression in response to different humid conditions, Chinese Spring seeds were sown in soil with a 12/12 h day/night and temperature regime of 25 °C/22 °C in an incubator. Seedlings were cultivated at 10–20% and 90–95% relative humidity (RH) conditions for three weeks. Thirty seedlings were subjected to each treatment and three parallel treatments were carried out for each humidity treatment. Leaves were harvested for analysis of *TaFAR* expression and cuticular waxes.

### Transcriptome sequencing, assembly and functional annotation

The seedling leaves and flag leaves from three wheat cultivars were harvested for total RNA extraction by RNAiso Plus reagent (Takara). The construction of cDNA libraries were accomplished using Illumina TruSeqTM RNA Sample Preparation Kit (Illumina, San Diego, USA) and after quality analysis by Agilent Bioanalyzer 2100 (Agilent Technologies, CA, USA), the libraries sequencing was performed on an Illumina HiSeq 2000 platform (Illumina, San Diego, CA) with paired-end sequencing technology. The clean reads were obtained by removing adaptor sequences, ambiguous ‘N’ nucleotides (with ‘N’ ratio over 5%) and low quality sequences from raw reads which were subjected to *de novo* assembly by Trinity software without reference genome^36^. To obtain sequence direction of the non-redundant unigenes, sequence similarity search was conducted against the NR, Swiss-Prot, KEGG and COG databases using the BLASTx algorithm (E-value < 10^−5^). The sequence direction of unaligned unigenes was predicted by ESTScan. For functional annotation, unigenes were subjected to NT, NR, Swiss-Prot, KEGG and COG databases using BLAST (v2.2.26) with an E-value cut off 10^−5^. Blast2GO was used to get GO annotation of unigenes and WEGO was used to obtain GO functional classification^37^. KEGG database was used for metabolic pathway annotation^38^.

### Cuticular wax extraction and chemical characterization

Wheat leaves and tomato fruits were harvested and immediately immersed in CHCl_3_ for 1 min at room temperature to extract the epicuticular and intracuticular waxes. Each sample was prepared by adding an internal standard, *n*-tetracosane (C24 alkane). The extracted wax mixtures were filtered and transferred to GC vials and evaporated to dryness under a stream of nitrogen gas. For gas chromatograph analysis, the dried wax samples were derivatized with 30 μl pyridine (Alfa Aesar) and 30 μl BSTFA [bis-N,O-(trimethylsilyl) trifluoroacetamide] (Fluka) for 60 min at 70 °C, then immediately dried under nitrogen gas before being re-dissolved in CHCl_3_ as described by Wang *et al.*^39^. Qualitative analysis of wax composition was performed using gas chromatograph equipped with mass spectrometric detector (GC-MS) (GCMS-QP2010, SHIMADZU, Japan). Each wax component was identified by comparing their mass spectra with those of authentic standards and literature data. Quantitative analysis was carried out using GC with flame ionization detector (GC-FID) (GC-2010Plus, SHIMADZU). Each wax component load was calculated based on FID peak areas, which were converted to mass units by comparison with the respective internal standard. The extracted surface areas of each sample were determined using digital images and ImageJ software. The wax content of leaf blade was calculated based on single-side leaf area.

For analysis of fatty alcohols, waxes or lipid mixtures were first separated by thin layer chromatography (TLC) silica gel (5 × 10 cm, silica gel, HSGF254, 0.15–0.20 mm) using CHCl_3_ as the mobile phase. Compound classes were then stained with primuline and were viewed under UV light. The distinct bands were scratched off and were analyzed by GC-MS and GC/FID as described above.

### Scanning electron microscopy (SEM) analysis

For the observation of epicuticular wax crystal, the samples were flattened and allowed to dry under 50–60 °C. About 0.5 cm segments were mounted onto scanning electron microscopy (SEM) stubs, and then coated with gold particles in an ion sputter coater (Hitachi E-1045, Japan). The coated samples were viewed with a Hitachi S-4800 field emission SEM using an accelerating voltage of 5 kV.

### Isolation of *TaFAR* genes from wheat leaf

Total RNA was extracted from leaves of Chinese Spring seedlings using Trizol (TaKaRa, Japan). First strand cDNA synthesis was performed using 1 μg of total RNA, oligo (dT)18 and PrimeScript® reverse transcriptase (TaKaRa) following standard protocols. The resulting cDNA mixture served directly as a template for the following PCR. The full-length cDNA sequences of putative *TaFAR*s were amplified using gene-specific primers labeled as FARx-RT-F and FARx-RT-R listed Supplementary Table S5. PCR was carried out with HS PrimeSTAR® DNA polymerase (TaKaRa). The corresponding cDNA fragments were separated by gel electrophoresis (1% agarose) and extracted using the DNA purification kit (TIANGEN, China). These fragments were subsequently ligated into pMD-18T vector (TaKaRa) and transformed into DH5α competent cells (TIANGEN) and sequenced. All subsequent constructs were made using this clone as template.

### Functional expression of TaFARs in Yeast

The coding sequences for *TaFAR2*, *TaFAR3* and *TaFAR4* were amplified using primers labeled as FARx-ORF-F and FARx-ORF-R in Supplementary Table S5. The amplified DNA fragments were cloned into the yeast expression vector pYES2 or pYES3 (Invitrogen) under the control of the GAL1 promoter to generate pYES2- or pYES3-TaFARx. For elongation of fatty acid chain lengths of yeast (*Saccharomyces cerevisiae*), a vector p416 MET25-FLAG3:Sur4-F262A/K266L with the mutated Sur4 (a yeast KCS) protein is used to obtain the mutated yeast INVSur4#^23^. The pYES3-TaFARx was co-transformed with p416 MET25-FLAG3:Sur4-F262A/K266L into wild-type INVSc1 strain (*MATa his3-D1 leu2 trp1-289 ura3-52*) cells. The pYES2-TaFARx alone was also transformed into INVSc1 cells. The corresponding empty vectors were used as positive control as detailed in Supplementary Table S3.

The yeast cells transformed with the different combinations or individual vector were cultured on synthetic complete (SC) selection medium agar plates lacking corresponding amino acid^24^. Then yeast cells were induced and lipids were extracted as described by Wang *et al.*^39^. The extracted lipid mixtures were purified by TLC and analyzed by GC-MS as described above.

### Overexpression of TaFARs in tomato and Arabidopsis *cer4-3* mutant

The *TaFAR2*, *TaFAR3* and *TaFAR4* DNA fragments containing the open reading frame sequences were cloned into plant expression vector pCXSN under the control of CaMV35S promoter using TA cloning method (Invitrogen)^40^. The constructs were transformed into *Escherichia coli* strain Top10 cell. The recombinant plasmid pCXSN-TaFARx and empty vector pCXSN were transferred to *Agrobacterium tumefaciens* strain GV3101 and then transformed into wild-type tomato (cv. MicroTom) with cotyledon as explants^41^. The homomycin-resistant plantlets were screened for the presence of *TaFAR* genes by PCR. T0 and T1 generation of transgenic tomato and control plants were grown in standard soil under ambient conditions in a greenhouse. The ripe fruits of T1 generation plants were used for wax analysis by GC-MS.

### Expression of TaFARs in Arabidopsis *cer4-3* mutant

Arabidopsis *cer4-3* mutants (SALK_038693C, ecotype Col-0) were transformed with pCXSN-TaFARs construct or empty vector control using *Agrobacterium tumefaciens*-mediated transformation and floral dipping as described by Clough & Bent^42^. Transformed plants were selected on hygromycin medium and then were transferred to soil and grown in a chamber at 22 °C with a long daylight cycle. F2 plants were screened for the presence of *TaFAR* genes by PCR. The rosette leaves and stems of F2 plants were used for wax analysis.

### Subcellular localization of TaFARs

For the expression of FAR-GFP fusion protein, the coding regions of three *TaFAR*s were cloned into a binary vector pA7-GFP with 35S promoter. The coding regions for *TaFARs* were amplified by the primers FARx-GFP-F and FARx-GFP-R listed in Supplementary Table S5. The amplified fragments were subsequently ligated into pA7-GFP, resulting in pA7-TaFARx-GFP. The target gene were fused with green fluorescent protein (GFP) and co-transformed into rice protoplasts with a known ER marker fused with cyan fluorescent protein (CFP)^43^. The imaging of transformed protoplasts was performed with LSM 7 Duo (Carl Zeiss, Germany). To avoid excitation crosstalk of CFP and GFP, the CFP was excited by 405 nm laser light, and the emission signal was collected up to 500 nm. The GFP was excited by 488 nm laser light and the emission signal was collected longer than 510 nm. Different fluorescent signals were collected via independent channel with a sequential mode.

### Expression of TaFARs in *Escherichia coli*

In order to express wheat TaFARs in prokaryotic expression system, the coding regions of *TaFAR2*, *TaFAR3* and *TaFAR4* were amplified using primers labeled as FARx-pET-F and FARx-pET-R in Supplementary Table S5. The amplified fragments were subcloned into pET-28a vector. The pET-TaFARs were transformed to *Escherichia coli* BL21, and expressions were induced by 0.4 mM IPTG for 6 h at 37 °C. Cells were harvested and separated by SDS-PAGE using 12% separate gels and 5% concentrated gels. The strong induced fusion protein bands were localized by staining with coomassie brilliant blue R250.

### Analysis of transcript level by quantitative real-time PCR

The RNA samples from different tissues and leaves under various stresses were used for cDNA synthesis by PrimeScript® reverse transcriptase following standard protocols. The quantitative real-time PCR (qRT-PCR) was carried out using SYBR^®^ Premix Ex TaqTM (TaKaRa) according to manufacturer’s instructions. Gene-specific primers FARx-qPCR-F and FARx-qPCR-R were designed to amplify a 187, 127 and 250-bp fragment for *TaFAR2*, *TaFAR3* and *TaFAR4*, respectively. Wheat actin gene (GenBank accession no. DQ115883) was used as an internal constitutively expressed control. Primers Actin-F and Actin-R were designed to amplify a 118-bp fragment. Each real-time PCR reaction was performed in 25 μl final volume on CFX96 real-time PCR detection system (BIO-RAD, USA) under the following program: 1 cycle of 30 s at 95 °C, followed by 40 cycles of 5 s at 95 °C, 30 s at 60 °C. All samples were repeated three times.

## Additional Information

**How to cite this article**: Wang, M. *et al.* Three *TaFAR* genes function in the biosynthesis of primary alcohols and the response to abiotic stresses in *Triticum aestivum*. *Sci. Rep.* **6**, 25008; doi: 10.1038/srep25008 (2016).

## Supplementary Material

Supplementary Information
