# Supplementary material for: Three TaFAR genes function in the biosynthesis of primary alcohols and the response to abiotic stresses in Triticum aestivum
Source: Sci Rep. 2016 Apr 26;6:25008. doi: 10.1038/srep25008 (PMC4845010; doi:10.1038/srep25008)
Supplement: Supplementary Information [file srep25008-s1.doc]

**Three *TaFAR* genes function in the biosynthesis of primary alcohols and the response to abiotic stresses in *Triticum aestivum***

**Meiling Wang^1,†^, Yong Wang^1,†^, Hongqi Wu^1^, Jing Xu^1^, Tingting Li^1^, Daniela Hegebarth^2^, Reinhard Jetter^2,3^, Letian Chen^4^ & Zhonghua Wang^1,*^**

**
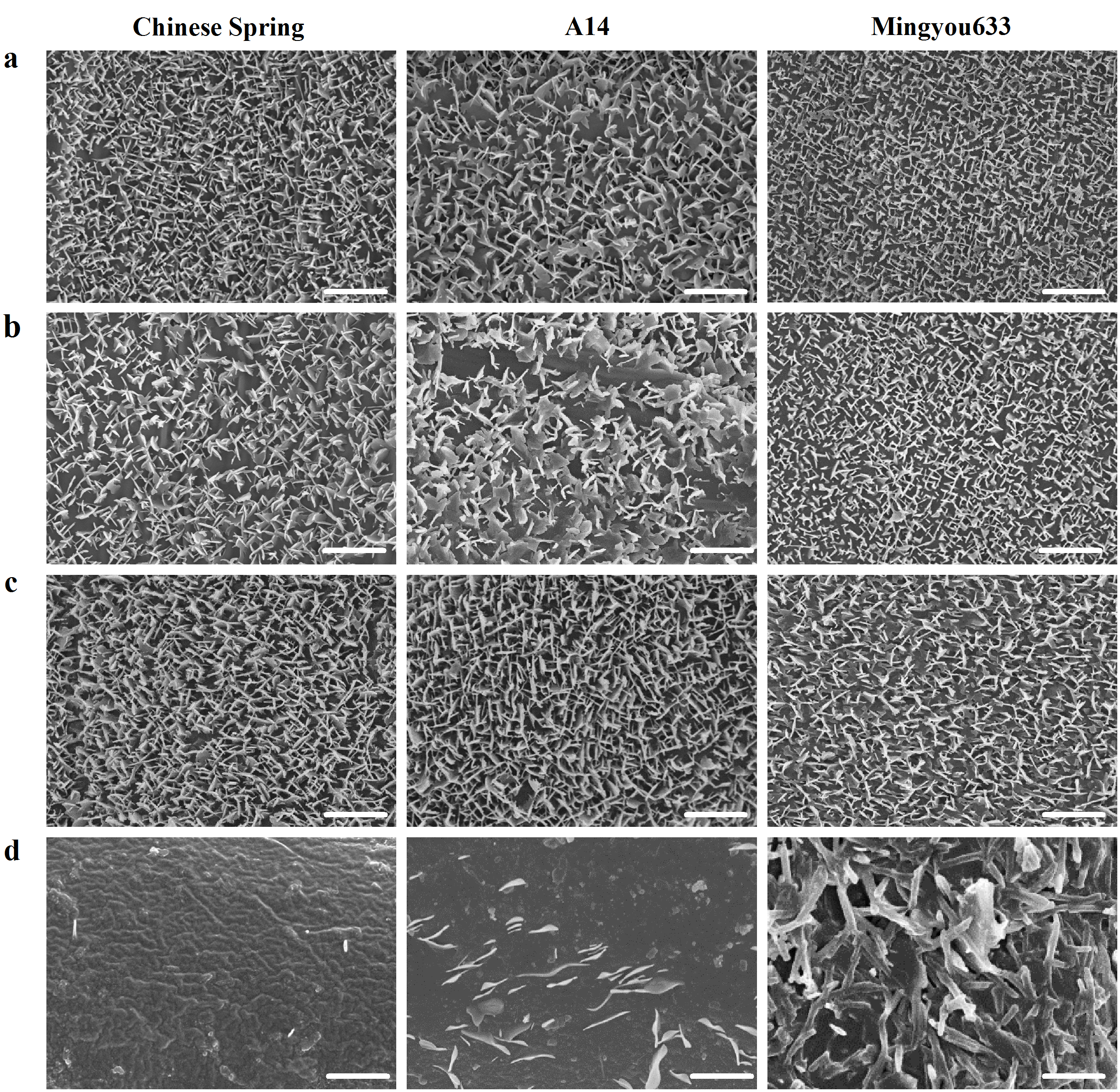
**

**Figure S1. Epicuticular wax crystals on wheat leaf surfaces detected by SEM.** The seedling leaves (**a,b**) and flag leaves (**c,d**) of three wheat cultivars were detected by scanning electronic microscope at 10,000× magnification. (**a,c**) Wax crystals on the adaxial surfaces of leaf blades. (**b,d**) Wax crystals on the abaxial surfaces of leaf blades. Scale bars = 2 μm.

**
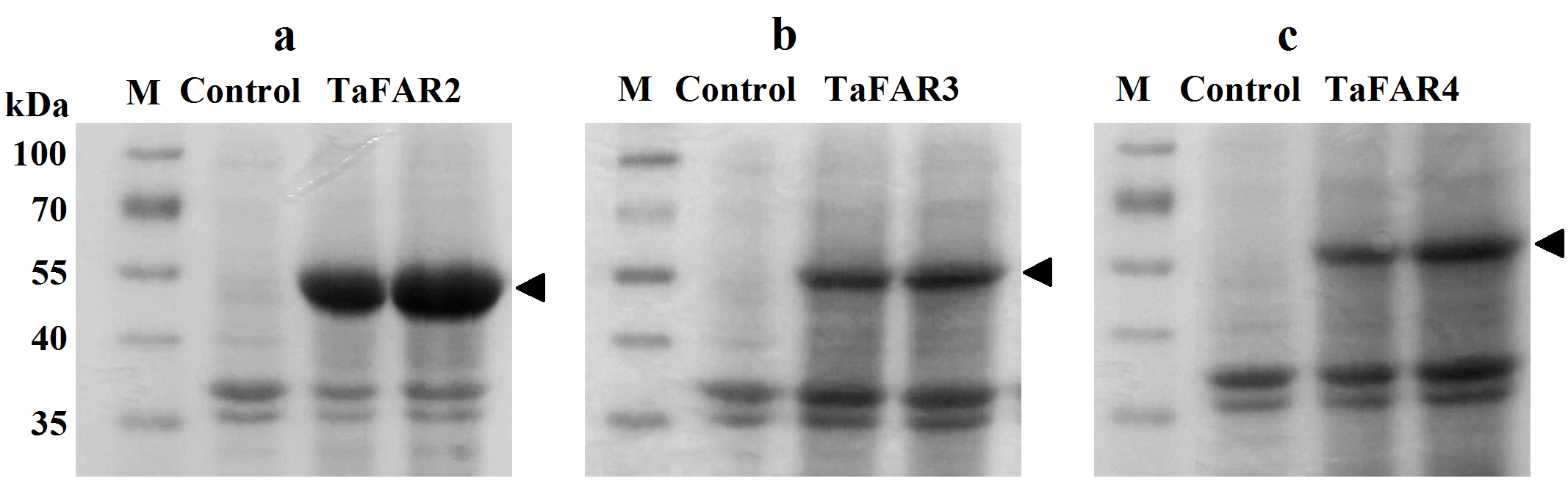
**

**Figure S2. Protein molecular mass analysis of TaFAR2 (a), TaFAR3 (b) and TaFAR4 (c) detected by SDS–PAGE**. Arrows indicate the His-TaFAR fusion proteins. The empty vector pET28a is as control. M, protein marker.


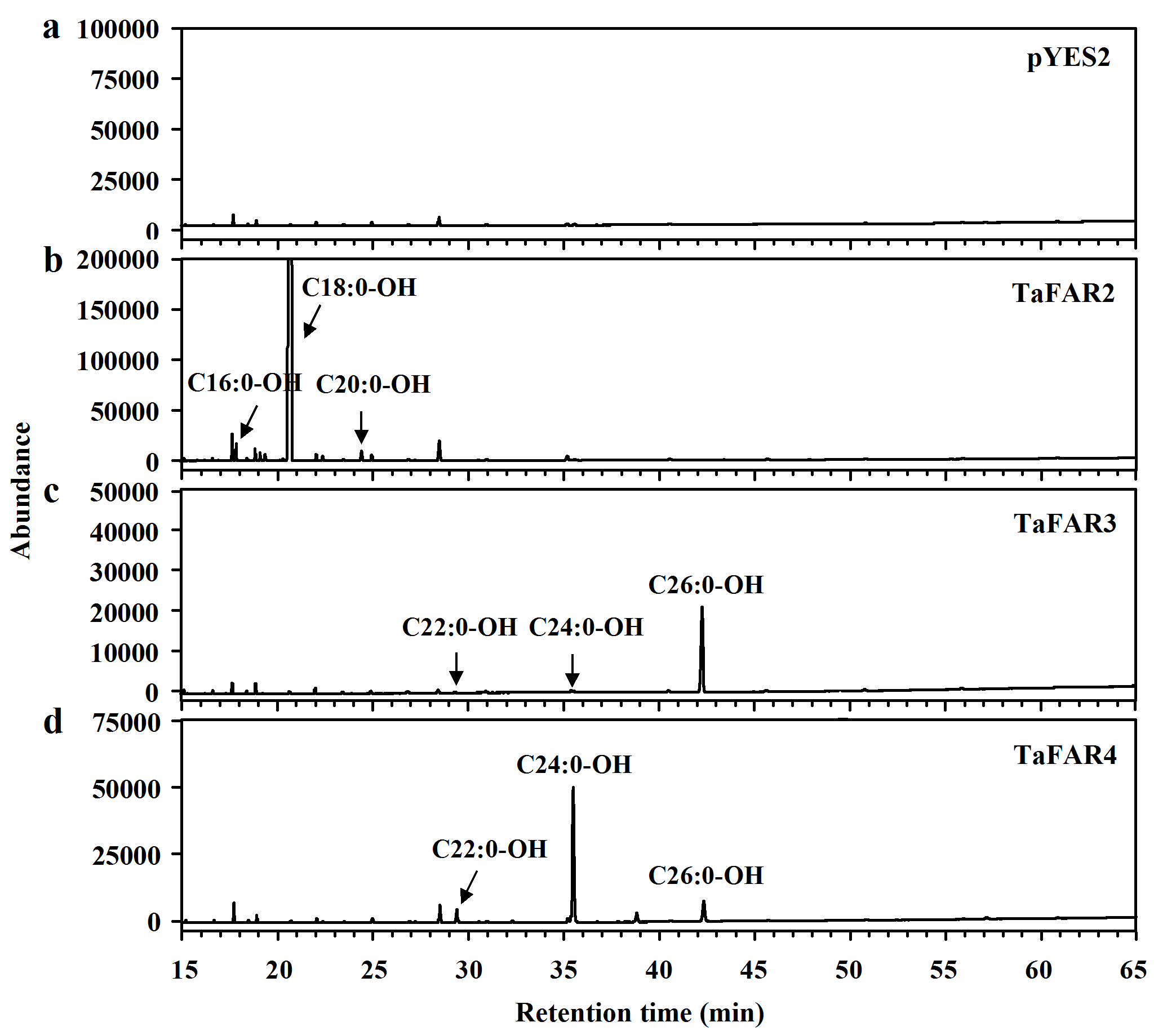


**Figure S3. Heterologous expression of three TaFARs in wild-type yeast.** The yeast expressing empty vector pYES2 (**a**) or with vector harboring TaFAR2 (**b**), TaFAR3 (**c**) or TaFAR4 (**d**). Transgenic yeast cells were grown in stringent medium lacking uracil. In empty vector control, no primary alcohols were detected. In contrast, yeast harboring TaFAR2, TaFAR3 and TaFAR4 constructs preferentially produced C18, C26 and C24 primary alcohols, respectively.


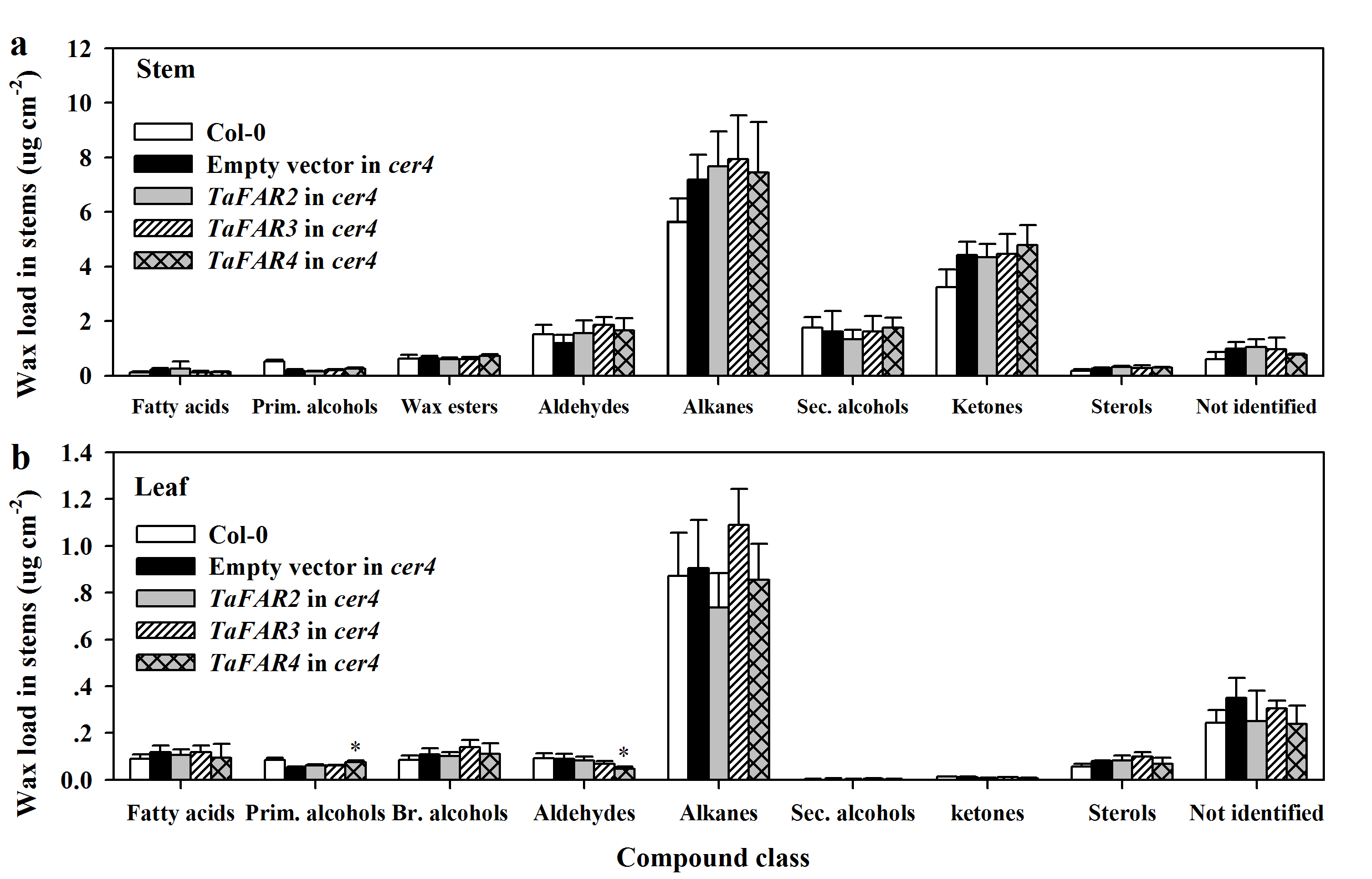


**Figure S4. The compound classes of cuticular wax in *Arabidopsis thaliana* lines expressing the three TaFARs.** Cuticular wax mixtures were extracted from stems (**a**) and leaves (**b**) of Arabidopsis transgenic lines expressing empty vector or vectors harboring *TaFAR* genes in *cer4* mutant background. Prim. alcohols, primary alcohols; Br. alcohols, Branched alcohols; Sec. alcohols, secondary alcohols. Values are the means of n≥5 replicates. Error bar = SD. An asterisk (*) represents significant differences between TaFAR-expressors and empty vector control value at *P* < 0.05.

**Table S1. Composition of cuticular waxes in wheat leaves.** The seedling leaves (SL) and flag leaves (FL) of three wheat cultivars were used for GC-MS analysis. The wax composition is shown as absolute amounts (μg cm^-2^ single-side leaf area). Prim. alcohols, Primary alcohols. Each value is the mean ± SD from three biological replicates. tr, trace, values that were below 0.01 μg cm^-2^.

| Chemical | Seedling stage | | |  | Heading stage | | |
| --- | --- | --- | --- | --- | --- | --- | --- |
| class | CS-SL | A14-SL | MY-SL |  | CS-FL | A14-FL | MY-FL |
| Total load | 9.70±0.75 | 10.50±0.12 | 11.55±0.99 |  | 12.03±0.94 | 14.02±0.88 | 16.81±0.66 |
| Fatty acids | 0.03±0.00 | 0.12±0.01 | 0.12±0.01 |  | 0.25±0.01 | 1.11±0.05 | 0.44±0.02 |
| Prim. alcohols | 8.00±0.77 | 8.77±0.19 | 9.82±0.72 |  | 7.42±0.52 | 7.31±0.60 | 6.64±0.13 |
| C20:0-OH | 0.05±0.04 | 0.05±0.00 | 0.05±0.01 |  | 0.09±0.03 | 0.05±0.01 | 0.03±0.01 |
| C22:0-OH | 0.09±0.02 | 0.07±0.01 | 0.06±0.02 |  | 0.10±0.02 | 0.07±0.01 | 0.04±0.01 |
| C24:0-OH | 0.13±0.02 | 0.05±0.00 | 0.02±0.00 |  | 0.54±0.11 | 0.60±0.12 | 0.58±0.09 |
| C26:0-OH | 0.33±0.04 | 0.51±0.06 | 0.18±0.04 |  | 0.41±0.05 | 0.58±0.07 | 0.31±0.09 |
| C28:0-OH | 7.26±0.67 | 7.80±0.14 | 9.22±0.63 |  | 6.04±0.40 | 5.73±0.62 | 5.33±0.20 |
| C30:0-OH | 0.11±0.07 | 0.21±0.03 | 0.23±0.06 |  | 0.19±0.08 | 0.22±0.05 | 0.24±0.03 |
| C32:0-OH | 0.03±0.04 | 0.08±0.04 | 0.06±0.04 |  | 0.05±0.01 | 0.06±0.03 | 0.11±0.06 |
| Esters | 0.05±0.00 | 0.07±0.01 | 0.07±0.03 |  | 0.12±0.04 | 0.12±0.02 | 0.11±0.01 |
| Aldehydes | 0.18±0.05 | 0.59±0.06 | 0.64±0.04 |  | 0.53±0.16 | 1.26±0.15 | 1.78±0.08 |
| Alkanes | 1.27±0.08 | 0.35±0.01 | 0.42±0.01 |  | 2.55±0.26 | 2.66±0.53 | 2.12±0.16 |
| β-diketones | tr | tr | tr |  | 0.39±0.19 | 0.88±0.08 | 4.36±0.66 |
| Not identified | 0.17±0.06 | 0.59±0.04 | 0.48±0.18 |  | 0.77±0.05 | 0.66±0.08 | 1.35±0.08 |

**Table S2. List of 32 alcohol-forming FARs and corresponding accession numbers.**

| No. | Gene ID | UniProt | Chromosome |
| --- | --- | --- | --- |
| 1 | Traes_4BS_CEA607A1D.1 | W5EA33 | 4BS |
| 2 | Traes_5AL_5785267FA.1 | W5EXB7 | 5AL |
| 3 | Traes_5DL_5597A11EC.1 | W5FTF7 | 5DL |
| 4 | Traes_4BS_BB8191175.1 | W5ED48 | 4BS |
| 5 | Traes_4AL_2475C298D.2 | W5DMC7 | 4AL |
| 6 | Traes_4AL_36D63D2CA.1 | W5DN29 | 4AL |
| 7 | Traes_4DS_849C911C9.1 | Q8L4V2 | 4DS |
| 8 | Traes_7DL_6DF800365.1 | W5I0F4 | 7DL |
| 9 | Traes_7DS_A3D9FFE80.1 | W5I9M6 | 7DS |
| 10 | Traes_4DL_021634BC0.1 | W5EET7 | 4DL |
| 11 | Traes_4BL_031666EE1.1 | W5E0Z8 | 4BL |
| 12 | Traes_5AL_DC104F3FE.1 | W5F232 | 5AL |
| 13 | Traes_4AS_BE9625780.1 | W5DZK5 | 4AS |
| 14 | Traes_7BS_2DA5DB033.1 | W5HT62 | 7BS |
| 15 | Traes_4DL_9480F40CF.1 | W5EJU6 | 4DL |
| 16 | Traes_4BS_DA955986B.2 | W5EDV0 | 4BS |
| 17 | Traes_7AS_63A586587.1 | W5HF56 | 7AS |
| 18 | Traes_7DS_93F9ED9BE.2 | W5I968 | 7DS |
| 19 | Traes_3AS_0EA847466.1 | W5CIS3 | 3AS |
| 20 | Traes_7BL_5F0CEA1BF.1 | W5HML2 | 7BL |
| 21 | Traes_3DS_71BF6EC00.1 | W5DHU4 | 3DS |
| 22 | Traes_7BL_FBA45F10E.2 | W5HS76 | 7BL |
| 23 | Traes3BF053100400CFD_t1 | D8LAK0 | 3B |
| 24 | Traes_4BL_304CCB4CB.2 | W5E2E6 | 4BL |
| 25 | Traes_3DS_44B20436F.1 | W5DGQ8 | 3DS |
| 26 | Traes_3AS_9B5BD6076.1 | W5CLZ3 | 3AS |
| 27 | Traes_7DS_CFC07E512.1 | W5IB09 | 7DS |
| 28 | Traes_1DS_EBA34AFC4.2 | W5ANI4 | 1DS |
| 29 | Traes_4AL_A10225940.1 | W5DS05 | 4AL |
| 30 | Traes_7AS_3FFD661DA.1 | W5HE25 | 7AS |
| 31 | Traes_7DS_A24610F5A.1 | W5I9K9 | 7DS |
| 32 | Traes_3AS_53B03CA17.1 | W5CKB1 | 3AS |

**Table S3. Transgenic yeasts.** The pYES2 or pYES2-TaFARx alone was transformed into wild-type yeast INVSc1 cells. The pYES3 or pYES3-TaFARx was co-expressed with the vector p416 MET25-FLAG3:Sur4-F262A/K266L into INVSc1cells.

| Genes expressed | Expression vectors | | Selection medium |
| --- | --- | --- | --- |
| empty vector | pYES2 |  | -Ura |
| TaFAR2 | pYES2:TaFAR2 |  | -Ura |
| TaFAR3 | pYES2:TaFAR3 |  | -Ura |
| TaFAR4 | pYES2:TaFAR4 |  | -Ura |
| SUR4# + empty vector | pYES3 | p416 MET25-FLAG3:Sur4-F262A/K266L | -Trp-Ura |
| SUR4# + TaFAR2 | pYES3:TaFAR2 | p416 MET25-FLAG3:Sur4-F262A/K266L | -Trp-Ura |
| SUR4# + TaFAR3 | pYES3:TaFAR3 | p416 MET25-FLAG3:Sur4-F262A/K266L | -Trp-Ura |
| SUR4# + TaFAR4 | pYES3:TaFAR4 | p416 MET25-FLAG3:Sur4-F262A/K266L | -Trp-Ura |

**Table S4. Chain length distributions of fatty alcohols in yeast expressing TaFARs.** Relative content (%) is given for each chain length of primary alcohol in each TaFAR-expressing yeast strain. Each value is the mean ± SD of three biological replicates. n.d., Not detected.

| Fatty acyl chain | Empty vector | TaFAR2 | TaFAR3 | TaFAR4 |
| --- | --- | --- | --- | --- |
| 16:0-OH | n.d. | 0.5 ± 0.2 | n.d. | n.d. |
| 18:0-OH | n.d. | 99.3 ± 0.2 | n.d. | n.d. |
| 20:0-OH | n.d. | 0.3 ± 0.0 | n.d. | n.d. |
| 22:0-OH | n.d. | n.d. | 2.4 ± 0.7 | 3.7 ± 0.2 |
| 24:0-OH | n.d. | n.d. | 1.0 ± 1.1 | 83.2 ± 0.8 |
| 26:0-OH | n.d. | n.d. | 5.7 ± 2.8 | 13.1 ± 0.6 |
| 28:0-OH | n.d. | n.d. | 89.8 ± 1.2 | n.d. |
| 30:0-OH | n.d. | n.d. | 1.2 ± 0.7 | n.d. |
| 32:0-OH | n.d. | n.d. | n.d. | n.d. |

**Table S5.** **Sequences of primers used in cloning and PCR reactions.** Restriction enzyme sites are bold.

| Primer name | Primer sequence |
| --- | --- |
| FAR2-RT-F | ATAATCTCACGAGCTCAGCTT |
| FAR2-RT-R | TTCAATTCTCACGCCAGATACTT |
| FAR2-ORF-F | CCC**AAGCTT**AAAAAAATGTCTATGGTGATCGGCGAAATGG |
| FAR2-ORF-R | AACCG**GAATTC**TCACGCCAGATACTTGAGCACACCAG |
| FAR2-GFP-F | CCG**CTCGAG**ATGGTGATCGGCGAAATG |
| FAR2-GFP-R | ACGC**GTCGAC**CGCCAGATACTTGAGCA |
| FAR2-pET-F | CCG**GAATTC**ATGGTGATCGGCGAAATG |
| FAR2-pET-R | ATAAGAAT**GCGGCCGC**TCACGCCAGATACTT |
| FAR2-qPCR-F | TGATCAAACATACCCAGATG |
| FAR2-qPCR-R | ATGATGACGACTGGAAGGTC |
| FAR3-RT-F | TTATTATCACGGTAGCTCGCGAT |
| FAR3-RT-R | CCTTTCTTTCTTATACGCTTCCCT |
| FAR3-ORF-F | CGG**GGTACC**AAAAAAATGTCTATGGACGCCAGAGCAGTGG |
| FAR3-ORF-R | ATAAGAAT**GCGGCCGC**TTATACGCTTCCCTTATTTTTGC |
| FAR3-GFP-F | CCG**CTCGAG**ATGGACGCCAGAGCAGTG |
| FAR3-GFP-R | ACGC**GTCGAC**TACGCTTCCCTTATTT |
| FAR3-pET-F | CGC**GGATCC**TTATTATCACGGTAGCTC |
| FAR3-pET-R | CCC**AAGCTT**GTTATACGCTTCCCTTAT |
| FAR3-qPCR-F | ATCCTTATTATCACGGTAGCTCG |
| FAR3-qPCR-R | CCTCAGTATCTTCTCAACCATCA |
| FAR4-RT-F | ATGGTTGACACACTGAGTGAAG |
| FAR4-RT-R | TCAGTTGAGCATGTGCTTTAG |
| FAR4-ORF-F | CCC**AAGCTT**AAAAAAATGTCTATGGTTGACACACTGAGT |
| FAR4-ORF-R | AACCG**GAATTC**TCAGTTGAGCATGTGCTTTAGGACC |
| FAR4-GFP-F | ACGAACGATA**CTCGAG**ATGGTTGACACACTGAGT |
| FAR4-GFP-R | GCTCACCATC**ACTAGT**ACGTTGAGCATGTGCTTTA |
| FAR4-pET-F | CGC**GGATCC**ATGGTTGACACACTGAGT |
| FAR4-pET-R | CCC**AAGCTT**GTCAGTTGAGCATGTGCT |
| FAR4-qPCR-F | CCTCTTCGGCCTCGACACCCTCT |
| FAR4-qPCR-R | CTAAAGCACATGCTCAACTGA |
| β-actin-F | GAGCTATGAGATGCCTGATGGT |
| β-actin-R | CGCTTCGTGTTACCAGGAACT |
